# Generating inner ear organoids containing putative cochlear hair cells from human pluripotent stem cells

**DOI:** 10.1038/s41419-018-0967-1

**Published:** 2018-09-11

**Authors:** Minjin Jeong, Molly O’Reilly, Nerissa K. Kirkwood, Jumana Al-Aama, Majlinda Lako, Corné J. Kros, Lyle Armstrong

**Affiliations:** 1 Institute of Genetic Medicine, Newcastle University, Newcastle, UK; 2 Sussex Neuroscience, School of Life Sciences, University of Sussex, Brighton, UK; 3 Department of Genetic Medicine and Princess Al-Jawhara Center of Excellence in Research of Hereditary Disorders, Faculty of Medicine, King Abdulaziz University, Jeddah, Saudi Arabia

## Abstract

In view of the prevalence of sensorineural hearing defects in an ageing population, the development of protocols to generate cochlear hair cells and their associated sensory neurons as tools to further our understanding of inner ear development are highly desirable. We report herein a robust protocol for the generation of both vestibular and cochlear hair cells from human pluripotent stem cells which represents an advance over currently available methods that have been reported to generate vestibular hair cells only. Generating otic organoids from human pluripotent stem cells using a three-dimensional culture system, we show formation of both types of sensory hair cells bearing stereociliary bundles with active mechano-sensory ion channels. These cells share many morphological characteristics with their in vivo counterparts during embryonic development of the cochlear and vestibular organs and moreover demonstrate electrophysiological activity detected through single-cell patch clamping. Collectively these data represent an advance in our ability to generate cells of an otic lineage and will be useful for building models of the sensory regions of the cochlea and vestibule.

## Introduction

Achieving the functions of the vertebrate inner ear requires a complex arrangement of cells that arise during embryonic development in a precisely orchestrated spatiotemporal manner. A principal cause of hearing loss is the death and/or dysfunction of the cells present in the organ of Corti^1–4^ which cannot regenerate post-partum in mammals meaning loss of individual cell types is irreversible^5^. This condition, known as sensorineural hearing loss, is a global healthcare challenge with 600 million persons worldwide affected^6^. Presbycusis, the age-related decline in hearing capacity is possibly the most prevalent neurodegenerative disease of ageing^7^ however chronic noise exposure and xenobiotic toxicity are significant contributing factors to hearing loss worldwide. The induction of human inner ear tissue from pluripotent stem cells could be applicable not only to modelling of sensorineural hearing loss but also for the generation of clinically useful sensory cells. Despite reports that progenitor cells capable of differentiating into cochlear hair cells may be isolated from neonatal mouse cochleae^8^ and putative differentiation of mesenchymal stem cells into hair progenitor cells^9^, the only cells that reliably differentiate into cells of an otic phenotype are pluripotent stem cells^10–15^. Most protocols have employed two-dimensional differentiation methods which are less likely to recapitulate inner ear development, therefore protocols that mimic the developmental progression towards inner ear construction are more likely to succeed in producing structures containing the desired cell types.

Recent work shows that pluripotent stem cells generate self-organising otic placode-like structures under 3D minimal culture conditions^16–19^ generating cells of the vestibular sensory epithelia, namely hair cells, neurons and supporting epithelial cells. To date, these protocols have not generated cells of a cochlear hair cell phenotype. Herein, we present a novel method that results in the conversion of hESC and hiPSC into 3D “organoids” containing otocyst-like structures comprising all the cell types normally present in the cochlea and vestibule.

## Results

### Adaptation of existing protocols for the generation of 3D otic organoids

We took advantage of a published protocol which utilised 3D culture conditions and stage-specific growth factor addition to generate otic organoids containing mechano-sensory hair cells^16^. We combined these conditions (Figure S1A) with forced aggregation of cells in U-shaped lipidure-coated plates (3000 cells/well) to direct differentiation of hESC however, this did not generate stable organoids (Figure S1B). Further modifications included substitution of GMEM for DMEM/F12 (Figure S1C) and increasing cell number per well in line with other literature protocols (Figure S1D)^20^, however only a concentration of 2-mercaptoethanol of 0.1 mM (Figure S2) was found to generate otic placode-like structures by day 32 of differentiation. Moreover, prior culture of hESC and hiPSC on mitotically inactivated mouse embryonic fibroblast feeder layers (MEFs) is essential for generation of otic organoids containing more mature cochlear cell types. The key points of this protocol are summarised as follows:

- Co-culture of hESC/hiPSC with MEF feeder layers prior to generation of embryoid bodies (EBs)
- Association of 9000 cells per well in 96-well lipidure-coated low adhesion plates to generate EBs
- Inclusion of the Rho-Kinase inhibitor Y-27632 (20 μM) and 0.1 mM 2-mercaptoethanol until differentiation day 8
- Addition of 1% matrigel to the differentiation medium between differentiation days 8 and 10.

### Characterisation of human pluripotent stem cell-derived pro-sensory otic vesicles

**Fig. 1:**
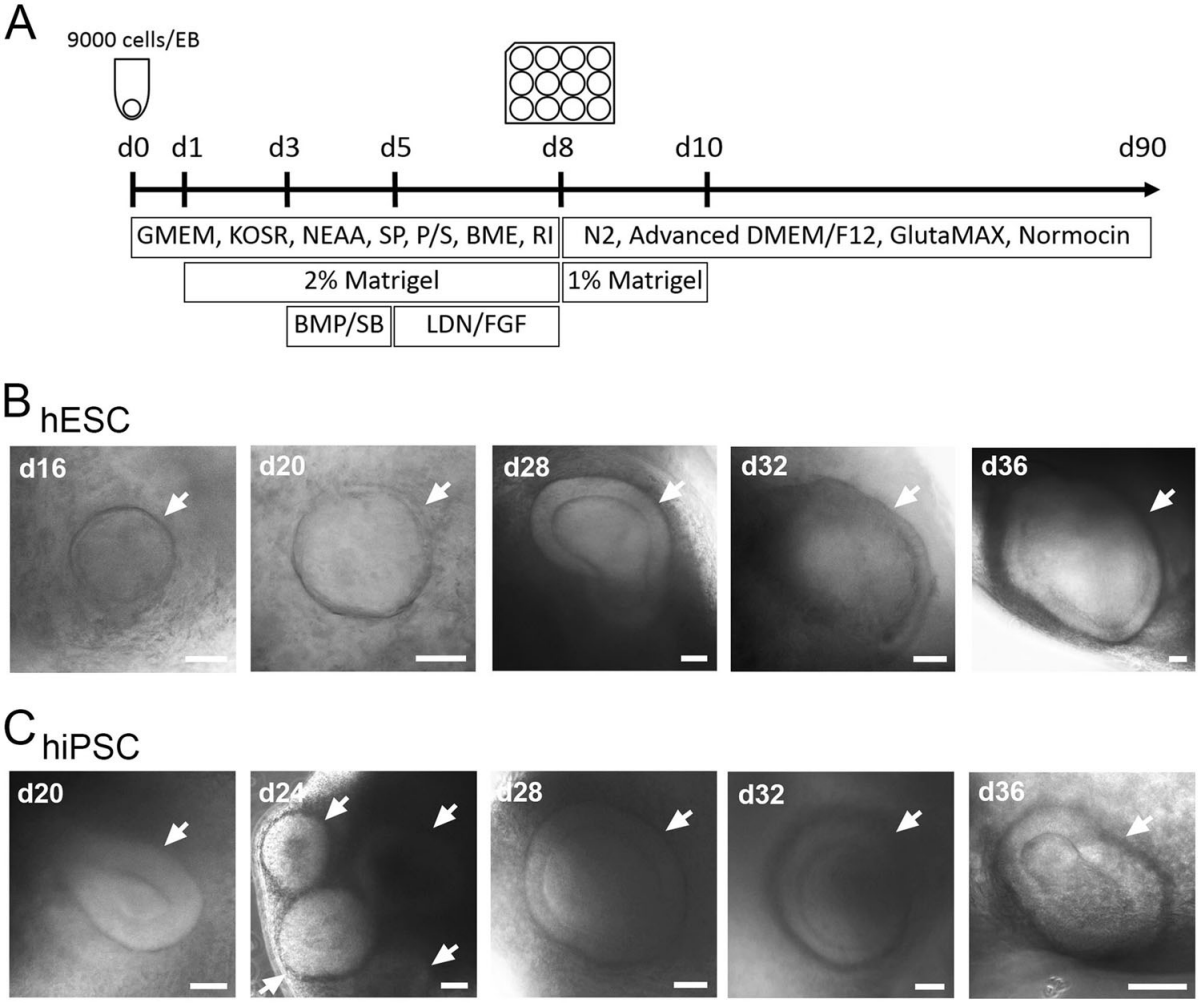
Generating otic organoids. **a** Schematic overview of the 90-day protocol used to generate otic organoids containing putative cochlear/vestibular hair cells and sensory neurons. GMEM Eagle’s Minimal Essential Medium, KOSR knockout serum replacement, NEAA nonessential amino-acids, SP sodium pyruvate, P/S penicillin/streptomycin, BME 2-mercaptoethanol, RI Y-27632, BMP BMP4, SB SB-431542, LDN LDN-193189, FGF FGF2. **b**, **c** Representative examples of 3D otic vesicles (indicated by white arrows) obtained between days 16 and 36 from hESC (H9) (**b**) and from hiPSC (SB-Ad3) (**c**). Pictures shown are representative of at least four independent experiments (*n* = 4). Scale bars represent 50 μm

Using our in-house protocol (Fig. 1a), we generated 3D organoids with vesicular structures (Fig. 1b, c) which were apparent from day 16 of differentiation, but became more numerous with time. By differentiation day 20, each organoid contained 1.5 ± 0.5 (±s.d., *n* = 32, 4 experiments) otic pit and otic vesicles from hESC and 1.6 ± 0.7 ( ±s.d., *n* = 32, 4 experiments) from hiPSC. The size range of vesicular structures was 100–200 µm.

**Fig. 2:**
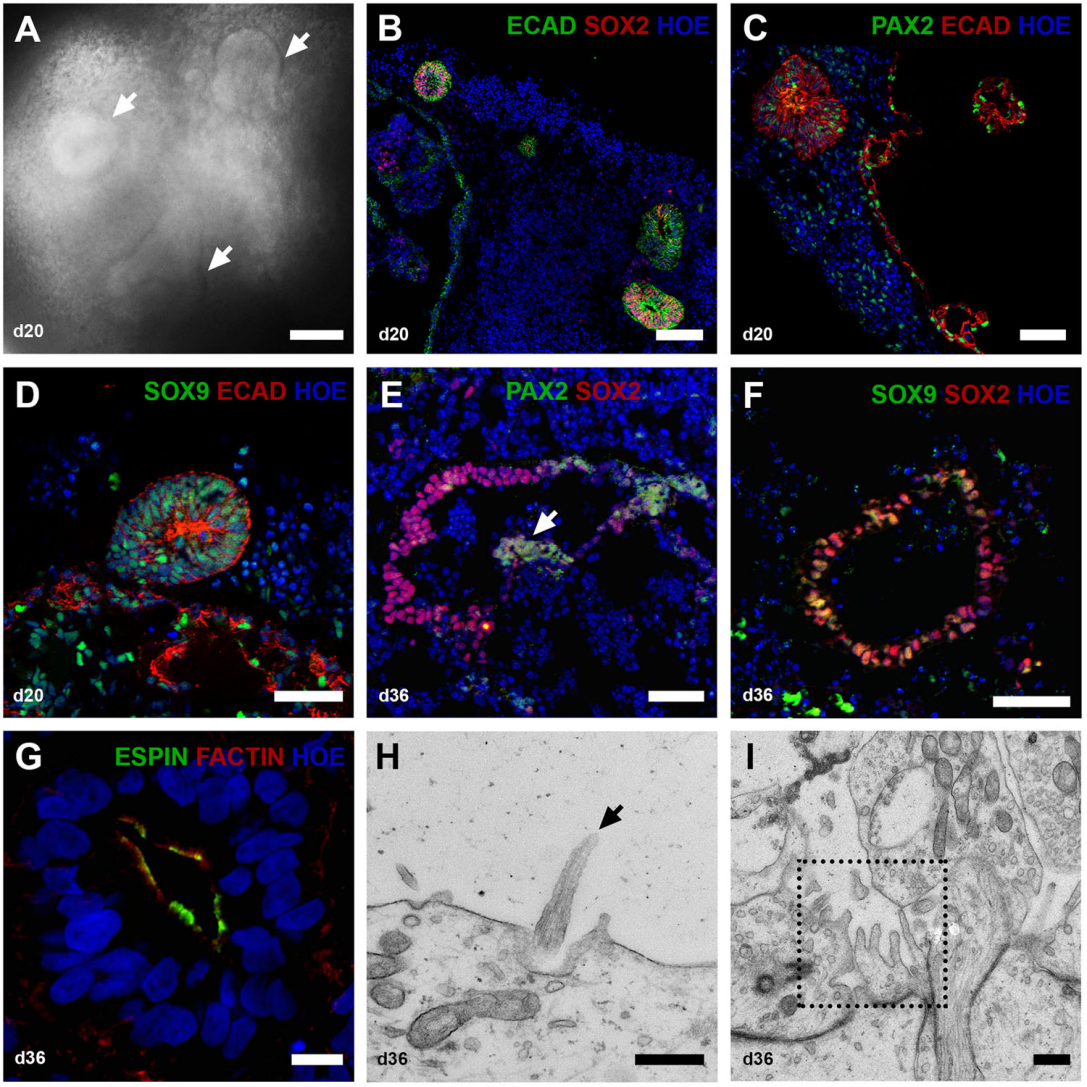
Characterisation of hESC-derived otic vesicles. **a** By day 20, the organoids contain otic vesicles (white arrows) in the size range 100–200 µm. (**b–d**) Immunohistological analysis of vesicular structures **b** ECAD^+^ SOX2^+^, **c** PAX2^+^ ECAD^+^, and **d** SOX9^+^ECAD^+^ in otic vesicles invaginating from the inner epithelium at day 20. Data are representative of 8–16 organoids from at least four separate experiments (*n* = 4). **e–g** Immunohistological analysis of prosensory otic vesicles (day 36). The vesicles contain prosensory cells expressing PAX2/SOX2 (**e**) and SOX9/SOX2 (**f**). Putative prosensory cells start to express ESPIN and F-ACTIN (**g**). Data are representative of 8–16 organoids from at least four separate experiments (*n* = 4). **h**, **i** TEM analysis of sections taken through the 36-day otic vesicles indicates the presence of immature kinocilia (**h**, black arrow) and microvilli (**i**, dotted square). Scale bars present 100 μm (**a**, **b**), 50 μm (**c**–**f**), 10 μm (**g**), 500 nm (**h**, **i**)

**Fig. 3:**
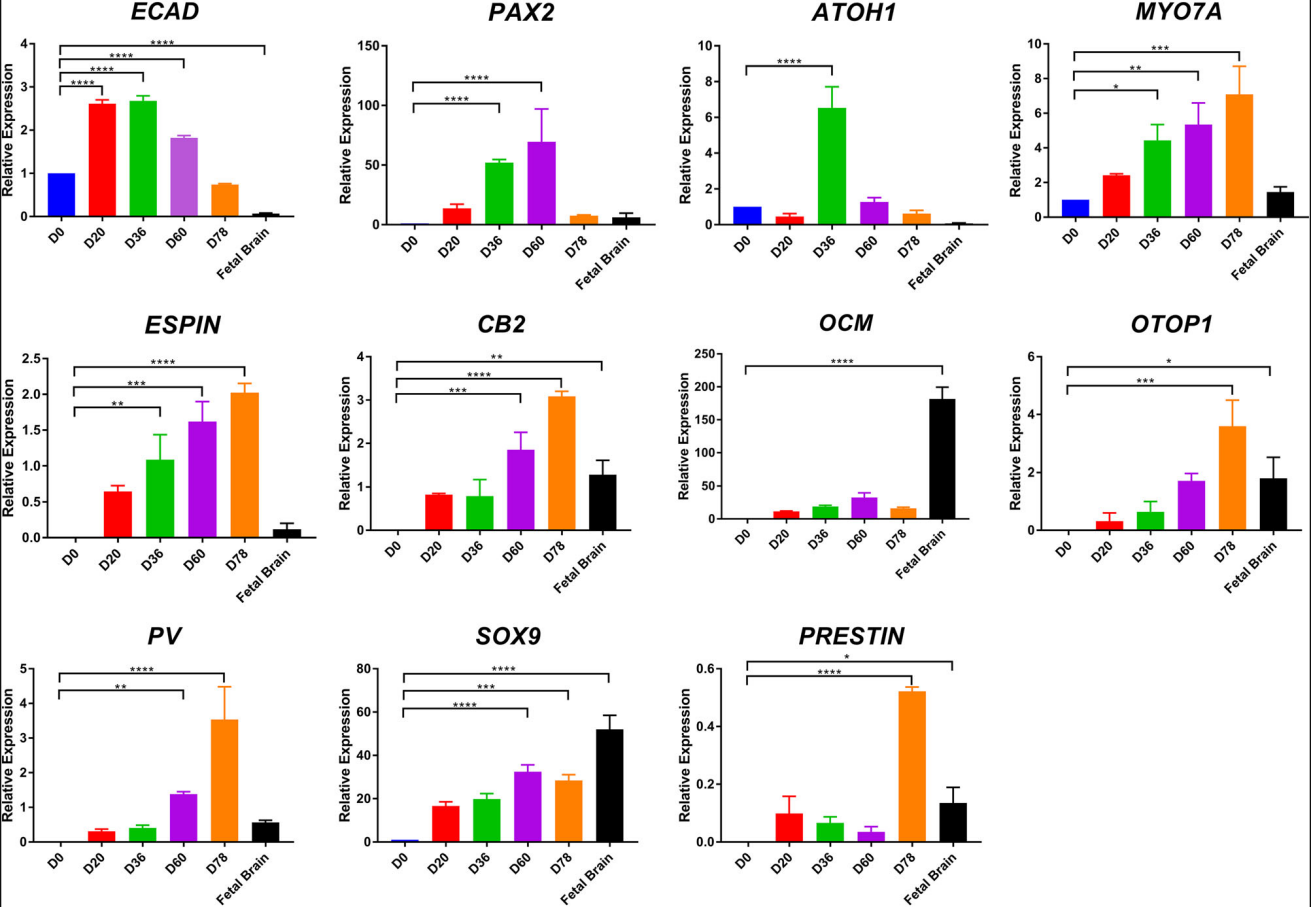
Quantitative PCR analysis during otic lineage differentiation of hESC. *ECAD*, *PAX2*, *ATOH1*, *MYO7A*, *ESPIN*, *CB2*, *OCM*, *OTOP1*, *PV*, *SOX9*, *PRESTIN* expression on day 0, 20, 36, 60, 78 (*n* = 3, *P < 0.05, **P < 0.01, ***P < 0.001, ****P < 0.0001; mean ± SEM)

Putative otic lineage cells in such vesicles were identified by double immunostaining with pro-sensory otic vesicle markers. In the E9.5 mouse, pro-sensory otic vesicles are defined by expression of Pax2, Pax8, Ecad, Sox2, Jag1, cyclin D1, and Myo7a^16^, but in equivalent human embryos (approximately 22 days post conception) the pro-sensory domains are only recognised by SOX2 expression^21^. Accordingly, we observed E-cadherin (ECAD)/SOX2-positive otic vesicles (Fig. 2b) corroborated by quantitative RT-PCR showing peak *E-cadherin* expression at differentiation days 20 and 36 (Fig. 3). Few cells within these otic vesicles expressed PAX2 (Fig. 2c) and SOX9 (Fig. 2d). Extra-vesicular PAX2 expression was also noted (Fig. 2c) and we speculatethese might be precursors of neurons that form in the 3D otic organoids. It is not clear that areas of cells expressing the above genes correspond to pro-sensory otic vesicles since no cells with sensory phenotype expression (such as MYO7A) are present at this stage and were not observed in day 20 organoids (data not shown). SOX2 expression quantified using Image J software on stained vesicle sections indicates both hESC (H9) and hiPSC (SB-Ad3) generated similar numbers of SOX2 expressing cells (25.27 ± 3.07% and 23.30 ± 1.78%; data representative of 8–16 organoids per experiment, *n* = 3), respectively.

By day 36, putative pro-sensory vesicles expanded to 300–450 μm. At this stage, otic vesicles were observed in 87.5 ± 45.1% from hESC and 30.6 ± 27.7% from hiPSC of organoids examined (24–36 organoids, *n* = 3). These pro-sensory vesicles show partial co-expression of PAX2/SOX2 (Fig. 2e) and SOX9/SOX2 (Fig. 2f) but are distinguished from day 20 vesicles by expression of F-ACTIN and the stereocilia marker ESPIN (Fig. 2g). Image J quantification indicates the percentages of SOX2 expressing cells present in day 36 vesicles to be 19.17 ± 1.49% (hESC) and 24.64 ± 6.67% (hiPSC; data representative of 16 organoids per experiment, *n* = 3). ESPIN expression at this time point is interesting since expression of this marker begins at 7 weeks of gestation in humans. The cells of the neuroepithelial layer that give rise to the vestibular sensory epithelium have a single micro kinocilium surrounded by microvilli^22^. A subset of these cells differentiates into vestibular hair cells which present several larger stereocilia^23^. TEM analysis of day 36 otic vesicles showed immature kinocilia (Fig. 2h) and microvilli (Fig. 2i). Similar observations were obtained by immunostaining of otic vesicles obtained from hiPSC (Figure S3B–G).

### Maturation and characterisation of human pluripotent stem-cell derived otic vesicles

**Fig. 4:**
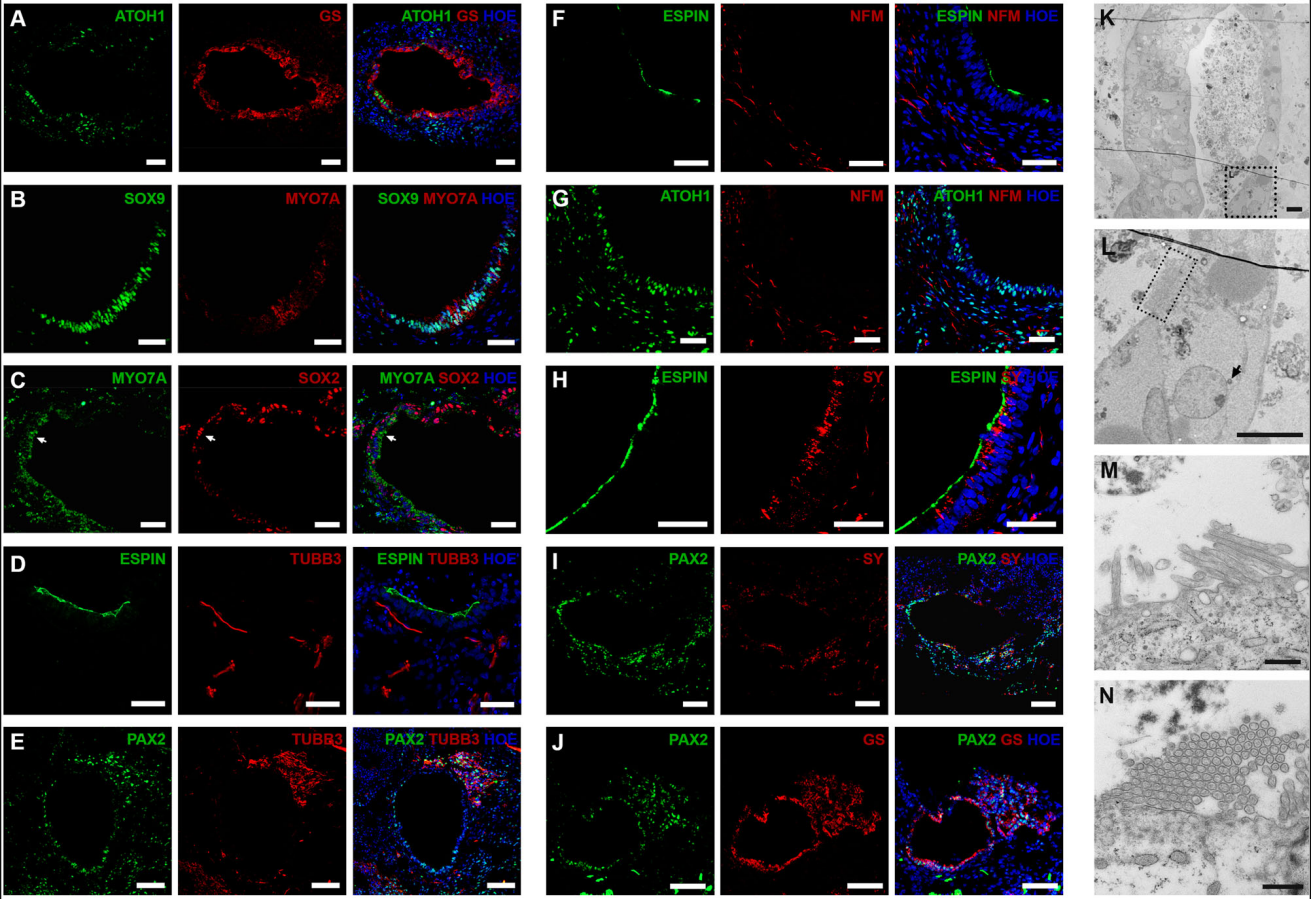
Immunohistological and TEM analysis of hESC-derived otic vesicles at day 60. ATOH1^+^ (**a**) and MYO7A^+^ (**b**, **c**) show possible hair cells underlying SOX9^+^ (**b**) or SOX2^+^ (**c**) supporting cells. TUBB3^+^ neurons extend to sensory epithelium expressing ESPIN (**d**) and PAX2 (**e**). NFM^+^ bipolar neurons present near the otic epithelium expressing ESPIN (**f**) and ATOH1 (**g**). SY^+^ synaptic neurons locate inside or near the sensory epithelium expressing ESPIN (**h**) and PAX2 (**i**). GS^+^ neurons innervate otic vesicles containing possible hair cells expressing ATOH1 (**a**) and PAX2 (**j**). Data are representative of 8–16 organoids from at least four separate experiments (*n* = 4). **k**–**n** TEM of the otic vesicles on day 60 (**k**). Higher magnification indicates putative pre-synaptic bodies (**l**, black arrow) and stereocilia (dotted square). Development of more mature stereocilia like projections (**m**) with stereocilia in cross section (**n**). Scale bars present 100 μm (**e**, **i**, **j**), 50 μm (**a**–**d**, **f**–**h**), 10 μm (**m**, **n**), 500 nm (**k**, **l**)

By day 60, 29.2 ± 8.3% of organoids from hESC and 30.6 ± 4.8% from hiPSC had otic vesicles (data representative of 36–48 organoids, *n* = 4) and expressed ATOH1 (Fig. 4a) and MYO7A (Fig. 4b, c) in cells adjacent to the lumen of putative otic vesicles. ATOH1 expression was observed in 62.85 ± 2.82% (hESC) and 48.11 ± 4.32% (hiPSC) of cells present. Quantitative RT-PCR showed peak ATOH1 expression at day 36 with stepwise increased expression of MYO7A from days 20–78 (Fig. 3). Some MYO7A expressing cells co-expressed SOX9 (Fig. 4b), but not SOX2 (Fig. 4c). The SOX2 expressing cells were found in a distinct layer from MYO7A, immediately below that adjacent to the lumen (Fig. 4c) and may be putative supporting epithelial cells. The numbers of SOX2-expressing cells in day 60 otic vesicles decreased slightly (19.01 ± 8.37% (hESC) and 12.67 ± 1.09% (hiPSC; data representative of 16 organoids per experiment, *n* = 3). Also apparent was the expression of proteins that indicate early innervation of prospective hair cells such as *β-III-tubulin* (TUBB3, Fig. 4d, e), *Neurofilament* (NFM, Fig. 4f, g), *Synaptophysin* (SY, Fig. 4h, i) and *Glutamine synthetase* (GS, Fig. 4a, j). TUBB3 was present in cells projecting towards the putative sensorineural epithelium expressing ESPIN (Fig. 4d) and PAX2 (Fig. 4e). Similarly, NFM staining together with ESPIN (Fig. 4f) and ATOH1 (necessary for hair-cell specification and differentiation)^24^ (Fig. 4g) suggested the presence of bipolar neurons, which are typical of inner ear ganglion neurons near the putative otic vesicles. Synaptophysin (SY), a post-synaptic marker of ribbon synapses^25^, was present in or near cells projecting into the putative sensory epithelium expressing ESPIN (Fig. 4h) and PAX2 (Fig. 4i). In vivo, glutamine synthetase GS is expressed by satellite glial cells which envelope the spiral ganglion neurons to maintain valid signal transmission^26^. We found strong GS expression in the epithelial cell layer adjacent to the otic vesicle lumen expressing ATOH1 (Fig. 4a) and PAX2 (Fig. 4j). These data indicate possible synaptic connections between putative neurons and hair cells. TEM suggested the presence of hair cells (Fig. 4k) with putative afferent nerve endings beneath the hair cells and a possible presynaptic body at their base (Fig. 4l). TEM images of day 60 organoids showed only a few short microvilli per cell (Fig. 4m, n), suggesting hair cell immaturity. Similar observations were obtained by immunostaining **(**Figure S4A–I**)** and TEM (Figure S4J, K) of hiPSC derived otic vesicles.

**Fig. 5:**
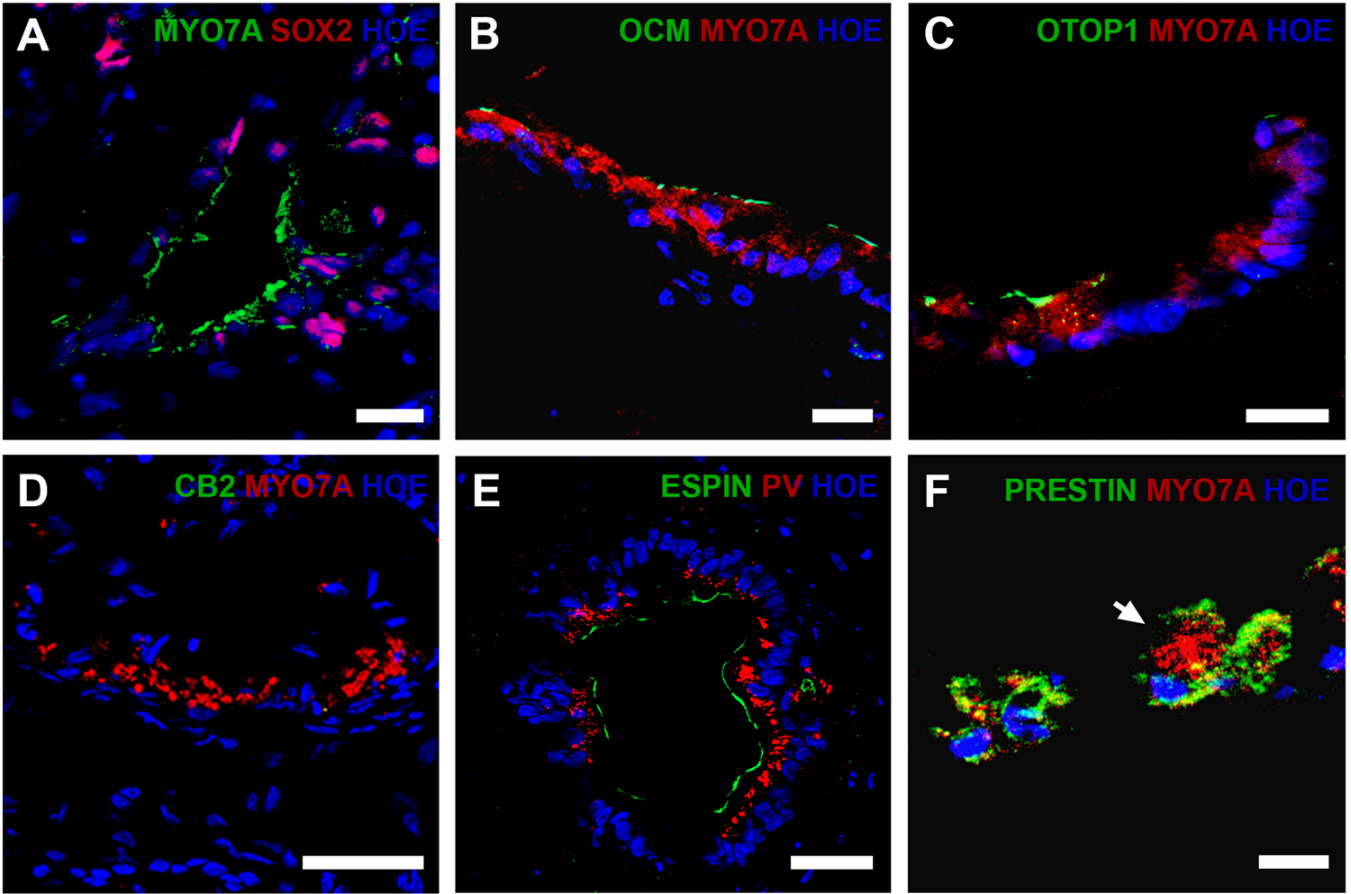
Immunohistological analysis of cochlear and vestibular hair cell markers at day 90. MYO7A^+^SOX2^-^ cells show the possibility of cochlear hair cells. **a** Presence of putative outer cochlear hair cells is indicated by expression of OCM^+^MYO7A^+^ (**b**) while OCM^−^MYO7A^+^ cells may be cochlear hair cells or type II vestibular hair cells. OTOP1^+^ cells are possible vestibular supporting cells (**c**); however, no cells with type II hair cell marker CB2 could be detected (**d**). Expression of PV cells indicates cochlear hair cells which also demonstrate ESPIN^+^ stereocilia (**e**) and PRESTIN^+^MYO7A^+^ cells (**f**) are supportive of an outer hair cell phenotype. Data are representative of 16 organoids from at least three separate experiments (*n* = 3). Scale bars present 50 μm (**d**), 40 μm (**e**), 20 μm (**a**–**c**, **f**)

By day 90, otic vesicles appeared more mature (Fig. 5) and were observed in 14.6 ± 4.2% from hESC and 22.2 ± 9.6% from hiPSC of organoids examined (36–48 organoids, *n* = 3). Published data suggest hiPSC differentiation only to hair cells of vestibular phenotype; hence we wished to determine if this also applied to our modified differentiation protocol. Four distinct populations of hair cells exist in the inner ear; type I and type II in the vestibular organs and inner (IHCs) and outer cochlear hair cells (OHCs). Expression of Sox2 and Pax2 may distinguish vestibular from cochlear hair cells in mouse and chicken, respectively^27,28^. We detected SOX2 expression in only a few putative hair cells (Fig. 5a), thus we analysed other potential markers to distinguish hair cell types. Oncomodulin (Ocm), specific to the cochlear OHCs of adult rats, mice and guinea pigs^29^, is a possible marker for putative OHCs. Ocm is presentin the hair cell body of mouse type I vestibular hair cells^30^; however, immunohistochemical location of the protein in our otic vesicles appears restricted to the cell membrane adjacent to the lumen (Fig. 5b) supporting an OHC phenotype. Otopetrin1 (Otop1) is specific to peripheral supporting cells of vestibular epithelia in mouse^31,32^. Some hESC/hiPSC derived hair cell-like cells marked by MYO7A expression (15.8 ± 2.43% in hESC and 12.9 ± 4.59% in hiPSC; 16 organoids per experiment, *n* = 3) also expressed OTOP1 near the apical surface of the sensory epithelium (Fig. 5c). In addition, calbindin2 (CB2) uniquely labels type II vestibular hair cells in mice^16^, however CB2 expression was undetected in the otic organoids (Fig. 5d). The expression of OTOP1 without CB2 suggests the presence of vestibular supporting cells but not type II vestibular hair cells. It has been reported that human IHCs and OHCs express PARVALBUMIN (PV);^33^ however whether PARVALBUMIN is localised to vestibular hair cells is unknown. Expression of PRESTIN uniquely labels human OHC basolateral plasma membrane^33^. On day 90, most hESC-derived ESPIN^+^ hair-cell-like cells expressed PARVALBUMIN (Fig. 5e) and some expressed PRESTIN (Fig. 5f) suggesting an OHC phenotype. Similar results were obtained using the hiPSC line (Figure S5A–F). Using 3D reconstructions, we estimated there were 46 ± 19 putative hair cells defined by MYO7A expression and putative 33 ± 18 outer hair cells defined by MYO7A and PRESTIN expression per hESC-derived otic vesicle. Similarly we estimated there were 71 ± 3 putative hair cells and 37 ± 14 outer hair cells (26 organoids, 3 experiments) per hiPSC-derived otic vesicles. This was corroborated by quantitative RT-PCR analysis showing hair cell markers (Espin, Prestin, Otopetrin 1, Parvalbumin) to be expressed at levels comparable or higher to a foetal brain sample at day 78 of differentiation (Fig. 3).

We observed cylindrical cells typical of type II vestibular or cochlear OHCs (Figure S6A and B) and cells with the more bulbous morphology of type I vestibular or cochlear IHCs (Figure S6C). More mature stereocilia-like structures were apparent (Figure S6D–F). The hair bundle length had increased from day 60 (~1–3 μm) and the longest stereocilia were 5–6 μm. At this stage, hair bundles were structurally similar to adult hair cells in vivo with cilia thickening towards the tip (Figure S6D) and arrangement of stereocilia into bundles (Figure S6D – G) some of which had the stereocilia arranged in a “stepwise” size order (Figure S6E) with rootlets extending into the cell body (Figure S6F). Moreover, cross-sections of possible stereocilia (Figure S6G) and kinocilia (Figure S6H) displayed the characteristic configuration of nine microtubule doublets surrounding two central microtubules^34^. Thin connections were observed between some stereocilia (Figure S6I) suggesting the formation of tip links which correlates reasonably well with the earliest appearance of such structures at gestation week 14^35^. Putative nerve endings beneath hair cell-like cells were observed (Figure S6J) with possible nerve endings filled with numerous and heterogeneous vesicles contacting potential hair cells (Figure S6K, L). In addition to button-like nerve terminals, a possible calyx terminal (enveloping the cell in a cup-like fashion) which contacts with type I hair cells was also observed (Figure S6M).

### Functional characterisation of human pluripotent stem cell-derived otic vesicles

**Fig. 6:**
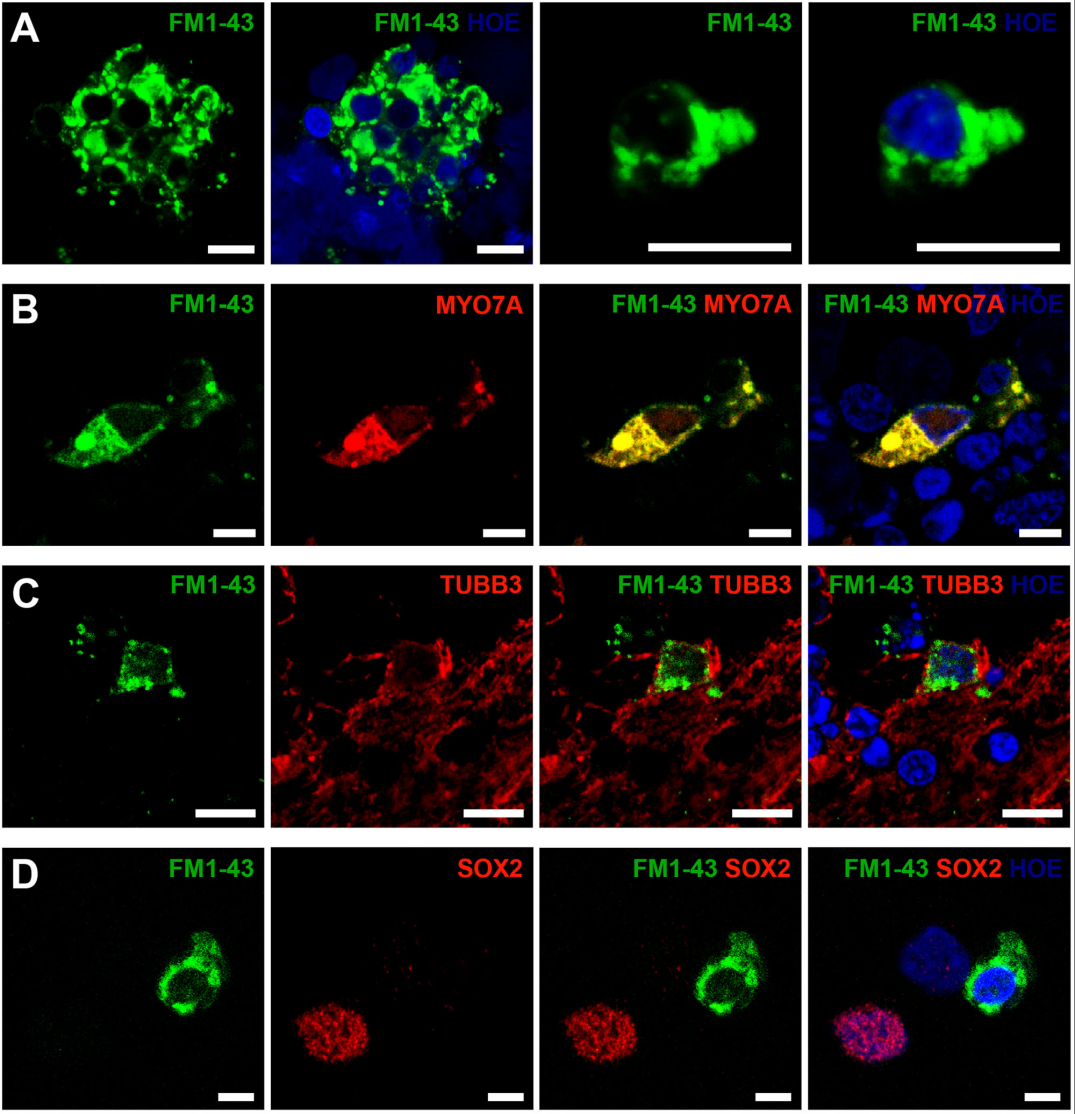
Functional properties of hESC-derived hair cells and neuronal cells using a FM1-43FX uptake assay at day 90. Confocal images of otic organoids dissociated to single cells and stained with FM1-43FX, indicated uptake of the dye by a subset of cells (**a**). FM1-43FX-labelled cells also express the hair cell marker MYO7A (**b**) and the neuronal marker TUBB3 (**c**), but not the supporting cell marker SOX2 (**d**). Data are representative of 8 dissociated organoids from at least three independent experiments (*n* = 3). Scale bars present 10 μm (**a**, **c**), 5 μm (**b**, **d**)

In vivo, mature hair cells exhibit rapid permeability to the styryl pyridinium dye FM1-43FX via mechano-transduction channels located in the stereocilia^36^. We show that staining with this dye is restricted to putative hair cells and beta-III-tubulin expressing putative sensory neurons in 3D otocyst-like structures (Fig. 6a–c). Co-immunostaining with otic lineage associated antibodies confirmed FM1-43FX localisation to cells with hair cell (Fig. 6b) or sensory neuron phenotype (Fig. 6c). FM1-43FX does not penetrate supporting epithelial cells and we observed a corresponding absence of staining in the SOX2 expressing cells (Fig. 6d). Again, these data are confirmed by parallel experiments using a hiPSC line (SB-Ad3) (Figure S7A–D).

**Fig. 7:**
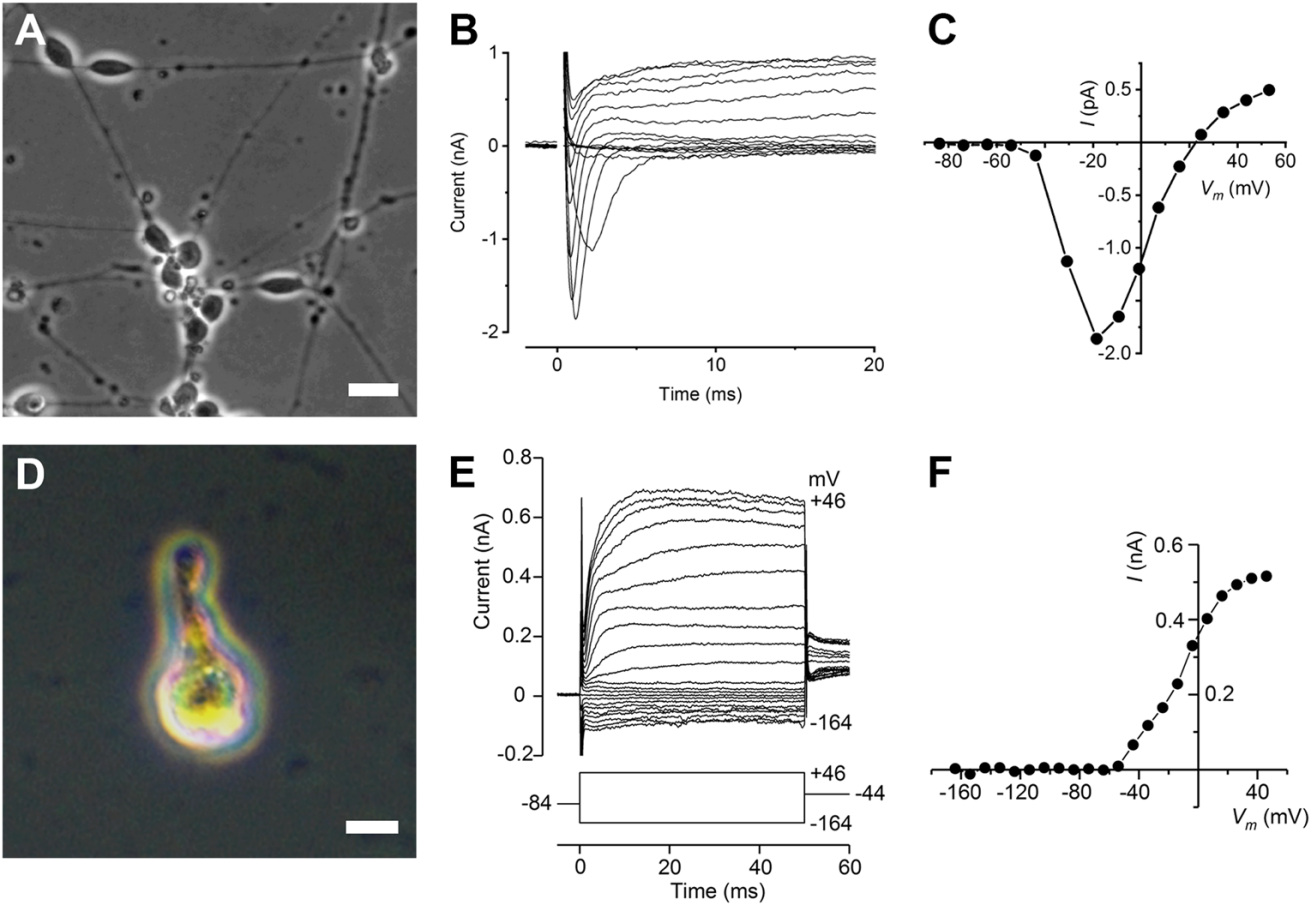
Membrane currents from cells with neuronal and hair-cell like morphology. **a**–**c** Bipolar neurons at the periphery of an otocyst from a day-90 hESC-derived organoid, 4 days after plating on a collagen substrate (**a**, phase contrast). Scale bar 20 μm. Voltage-clamp recordings show rapid inward Na^+^ currents followed by slower outward K^+^ currents (**b**). Peak *I*–*V* curve derived from **b** (**c**), predominantly determined by the inward Na^+^ currents. Currents, averaged from three repetitions of the stimulus protocol, were elicited from a −84 mV holding potential in nominal 10 mV steps. Recording conditions: *C*_m_ 6.6 pF; *R*_s_ 3.2 MΩ; *g*_leak_ 3.2 nS. Holding current of −48 pA was set to zero, leak currents were subtracted and membrane potentials were corrected for voltage drop across *R*_s_. **d**–**f** Recordings from a cell with hair cell-like morphology. Single hair cell isolation detected by brightfield microscopy using a Zeiss Axiovert 200 M (**d**). Scale bar 10 μm. Voltage-clamp recordings (**e**) showing currents (averaged from three repetitions) elicited by hyperpolarising and depolarising voltage steps in 10 mV nominal increments from the holding potential of −84 mV, covering a range from −164 to +46 mV, before returning to a constant potential of −44 mV (shown schematically below the current traces). *I*–*V* curve (**f**) derived after subtracting linear leak currents from recordings shown in **a**, plotting maximum currents near the end of the voltage steps. Recording conditions: *C*_m_ 4.2 pF; *R*_s_ 3.9 MΩ; *g*_leak_ 1.1 nS. Holding current of 5 pA was set to zero; membrane potentials were not corrected for voltage drop across *R*_s_ as errors were <2 mV

We examined the electrophysiology of prospective neurons and hair cells by culturing dissected organoids on collgen coated coverslips (because 3D organoids are too dense to patch clamp individual hair cells) (Fig. 1b). Four days after plating, neuronal-like cells appeared at the periphery of the otic vesicles (Fig. 7a) from which it was possible to obtain electrophysiological recordings. All cells examined in voltage-clamp displayed a fast-activating, rapidly inactivating inward current, presumably a Na^+^ current, in response to increasing membrane depolarisation. Rapid inward currents are followed by slower outward K^+^ currents (Fig. 7b). The peak *I*–*V* curve derived from the voltage-clamp recordings is predominantly determined by the inward Na^+^ currents (Fig. 7c), which activated positive to about −45 mV and reached a maximum size of −1.86 nA at −18 mV. A further three cells with similar morphology were tested with a Cs^+^-based intracellular solution, which blocked the outward K^+^ currents. In these cells (*C*_m_ 8.1 ± 1.1 pF), peak inward Na^+^ currents averaged −1.35 ± 0.05 nA at −15.3 ± 1.8 mV (all means ± SEM). The expression profile of membrane currents in neuron-like cells closely resembled that of primary auditory neurons in the rat cochlea^37^. Although these data were interesting, it is possible that the neuronal outgrowths do not originate from putative spiral ganglion neurons present in the otic vesicles. To circumvent this problem, we disaggregated dissected otic vesicles to single cells, then plated the cell suspension onto growth factor reduced matrigel coated coverslips. Adherent cells were identified as hair cell-like (Fig. 7d) or neuronal-like after 4 days on the basis of morphology and FM1-43FX staining allowing us to record small voltage-gated K^+^ currents, while Na^+^ currents were absent. Figure 7e shows the time course and size of the currents for one cell. The *I*–*V* curve for this cell, obtained after subtraction of the leak conductance, shows the K^+^ currents start to activate positive to about −55 mV (Fig. 7f). The outward K^+^ current reached a size of 0.33 nA at −4 mV for this cell, while for another similar cell the size was 0.20 nA (Fig. 7f).

## Discussion

We report an optimised protocol for generating putative cochlear hair cells from hESC and hiPSC. Within days 16–20, putative otic vesicles started to form from epithelium situated on the surface of the cell aggregates. Approximately 20 days later, ESPIN-positive microvilli appeared within possible pro-sensory otic vesicles progressing toMYO7A expressing hair-cell-like cells appeared and in parallel, neuronal-like cells developed alongside otocysts containing primitive hair cells by day 60. On day 90, otic organoids included PRESTIN positive potential OHCs and amphora-shaped potential vestibular type I hair cells. Differing propensities of the single hESC and hiPSC lines used in this study to generate otic vesicles containing more mature cell types only became apparent at differentiation day 90. This may reflect differences in the genetic background of the individual pluripotent stem cell lines, or an intrinsic difference between embryonic and induced pluripotent stem cells, however it is difficult to make this conclusion until the differentiation protocol is applied to broader range of pluripotent stem cell lines. Quantitative RT-PCR analysis, immunohistochemistry, TEM, and the capability to take up FM1-43FX support the development of both vestibular and cochlear hair cell phenotypes in addition to possible innervation by putative neurons. Our electrophysiological analysis indicates some similarity to immature hair cells from embryonic mice but this does not allow us to confirm their identity as either cochlear or vestibular hair cells. That notwithstanding, the modest differences between our protocol and that of the Hashino group may be sufficient to induce the formation of hair cells with a cochlear phenotype. They key modifications employed in our protocol are the prior culture of hESC and hiPSC on MEFs and the concentration of 2-mercaptoethanol needed to ensure robust conversion of embryoid bodies into otic organoids. While the requirement for a precise level of 2-mercaptoethanol can be understood in terms of modulating concentrations of oxygen free radicals such that survival of some cell types may be favoured, the need for MEF co-culture is less easily rationalised. Culture in matrigel/mTeSR1 is known to cause lineage-specific priming of hESC that enhances neural differentiation at the expense of other differentiation pathways such as haematopoiesis^38^. Exposure of mTeSR1 primed hESC to MEFs and/or MEF conditioned hESC medium reverses this preference so it is possible that a similar mechanism operates in the requirement for MEF co-culture prior to otic differentiation observed in this study. A key question arising from previous studies is the mechanism by which differentiation into vestibular hair cells becomes the “default” phenotype. Vestibular hair cells appear earlier during development^39–41^ so perhaps preference for vestibular differentiation reflects this timescale in vitro, but given that the 3D organoids from our study and others have been maintained for up to 150 days in culture, this seems a lesser possibility. Sonic hedgehog (Shh) is required for terminal differentiation of cochlear hair cells^42,43^. This process seems to require Shh synthesis by the developing spiral ganglion neurons, but expression levels decrease from a basal to apical direction in the developing cochlea and thispattern matches that of hair cell differentiation in the cochlear duct^44^. Thus temporal expression levels of Shh may differ in 3D organoids produced using our protocol or this may even be a consequence of the different pluripotent stem cell lines used in our study. Mechano-sensory cell specification is mediated by FGF20 signalling andt this may be specific to the cochlea since formation of vestibular structures is unaffected in Fgf20 knockout mice^45^. A role for FGF signalling in otic development is known and the ligands involved in otic placode induction have been identified as FGF3 and FGF10^46,47^. In our study, organoids were exposed to FGF2 on days 5–8 to promote otic lineage differentiation; however, exposure to other FGFs and optimisation of relative exposure times may be beneficial for generation of otic vesicles containing greater numbers of cochlear hair cells.

Regardless of the efficiency of differentiation, target cell maturity is still an issue. Despite identification of synaptic vesicles, cells with these were a minority and if Shh signalling from developing spiral ganglion neurons is a prerequisite for cochlear hair cell terminal differentiation, putative hair cells in our otic vesicles may not be as mature as in the adult organ of Corti. The small size of the K^+^ currents recorded from these cells also correlates with that seen in cochlear inner and outer hair cells in early postnatal mice^48–50^. Consistent with this possibility is the observation of limited numbers of stereocilia connected by putative tip links or cross-links. Immaturity of somatic cells differentiated from pluripotent stem cells has been reported^51,52^. For iPSC-derived cardiomyocytes, mechanical and or electrophysiological stimulation enhances their structural maturity in vitro^53^ so it is conceivable that cochlear hair cells developing within otic organoids may require acoustic stimulation to become fully functional. This is consistent with the functional maturation of cochlear hair cells and the development of hearing in neonatal mice^54^, however to improve the utility of our inner ear organoid culture to the point where we can investigate the possible contribution of acoustic stimulation, we need to manipulate additional signalling molecules that could amplify the otic induction process. This could focus on various FGFs or retinoic acid (known to contribute to the control of location-specific aspects of hair cell phenotypes)^55,56^ but a more tractable approach might be to extend the development of fluorescent reporter systems analogous to the ATOH-GFP cell line developed by Koehler et al.^19^

In conclusion, the studies in this project describe for the first time the possible differentiation of hESC and hiPSC to both cochlear and vestibular hair cells and neurons using a 3D organoid system.

## Materials and methods

### hESC and hiPSC culture

hESC (H9) and hiPSC (SB-Ad3) on Matrigel hESC-qualified matrix (BD; www.bd.com) were cultured in mTeSR1® medium (StemCell Technologies; www.stemcell.com). hESC and hiPSC colonies were treated with 0.02% EDTA (Lonza; www.lonza.com) and picked out by mechanical disruption using a tip to give rise to small clumps and plated on the Matrigel®-plate. hESC and hiPSC were maintained under mTeSR1® medium and seeded on a feeder layer of mouse embryonic fibroblasts (MEF) (mitotically inactivated by irradiation) for experimentation until passage 60. hiPSCs were used before passage number 50. Medium for the cells on MEF is knockout-Dulbecco’s modified Eagle’s medium (DMEM), 1 mM l-glutamine, 100 mM nonessential amino acids (NEAA), 20% knockout serum replacement (KOSR, Gibco; www.lifetechnologies.com), 1% penicillin–streptomycin (Gibco) and 8 ng/ml bFGF (Invitrogen; www.lifetechnologies.com). The medium was changed daily.

hESC and hiPSC on MEF were dissociated with Accutase (Gibco; www.lifetechnologies.com) for 3 min, resuspended in differentiation medium and plated 100 μl per well (9000 cells/well) on 96-well low-cell-adhesion U-bottom plates (Lipidure Coat, NOF; www.nofamerica.com). Differentiation medium was G-MEM (Life Tech; www.lifetechnologies.com) supplemented with 1.5% KOSR (Invitrogen; www.lifetechnologies.com), 0.1 mM NEAA, 1 mM sodium pyruvate, 1 mM penicillin/streptomycin and 0.1 mM 2-mercaptoethanol (Life Tech). On day 1, half of the medium in each well was exchanged for fresh differentiation medium containing Growth Factor Reduced Matrigel (2% (v/v) final concentration, BD; www.bd.com). On day 3 of the protocol, BMP4 (10 ng/ml, Gibco) and SB-431542 (1 μM, TOCRIS; www.tocris.com) were added to each well at 5× concentration in 25 μl of fresh media. On days 5, FGF2 (25 ng/ml, Gibco) and LDN-193189 (1 μM, STEMGENT; www.stemgent.com) were added to each well at 6X concentration in 25 μl of fresh media. The concentration of Y-27632 (Chemdea) was maintained at 20 μM throughout days 0–8. The concentration of Matrigel was maintained at 2% (v/v) throughout days 1–8. On day 8 of differentiation, organoids were transferred to 6 or 12 well plates (Lipidure Coat, NOF) in N2 medium containing 1% (v/v) Matrigel. N2 medium contained Advanced DMEM/F12 (Gibco), 1X N2 Supplement (Life Tech), 50 μg/ml Normocin (Invitrogen) and 1 mM GlutaMAX. After 48 h the medium was changed completely with new N2 medium. Beginning on day 10, half of the medium was changed every other day during long-term floating culture for up to 90 days.

### Quantitative RT-PCR (qRT-PCR)

RNA was isolated using the ReliaPrep™ RNA Cell Miniprep System (Promega; https://www.promega.co.uk). Single-stranded complementary DNA was synthesised from 1 μg of the RNA samples using the GoScript Reverse Transcription System (Promega). The qRT-PCR was performed with the GoTaq qPCR Master Mix (Promega) in a QuantStudio™ 7 Flex real-time PCR system (Life Technologies). The reaction parameters were as follows: 95 °C for 2 min to denature the cDNA and primers, 50 cycles of 95 °C for 15 s followed by annealing/extend 60 °C for 60 s. A comparative Ct method was used to calculate the levels of relative expression, whereby the Ct was normalised to the endogenous control glyceraldehyde-3-phosphate dehydrogenase (GAPDH). This calculation gave the ΔCt value, which was then normalised to a reference sample (i.e. a positive control), giving the ΔΔCt. The fold change was calculated using the following formula: 2^−ΔΔCt^. Foetal brain (BioChain; https://www.biochain.com/) was used for positive control.

Primers used: *ECAD* forward: AGCGTGTGTGACTGTGAAGG, reverse: CTCTTCTCCGCCTCCTTCTT; *PAX2* forward: GAGCGAGTTCTCCGGCAAC, reverse: GTCAGACGGGGACGATGTG; *ATOH1* forward: CCTTCCAGCAAACAGGTGAAT, reverse: TTGTTGAACGACGGGATAACAT; *MYO7A* forward: GAGTCAGGCTTCCTCAGCTT, reverse: GTGACCAGGGCCACAATCTC, *ESPIN* forward: CAGAGTGCAGGACAAAGACAA, reverse: GCAGCGTAGTGGATAGGCAG; *CB2* forward: AGTGTTGGCTGTGCTCCTCATC, reverse: GTTGATGAGGCACAGCATGGAG; *OCM* forward: CGGTTCATAGACAACGACCAGAG, reverse: CATTATCCGCCGCAGCCATCAA; *OTOP1* forward: ATCCACTCGGTGTTCACCAACC, reverse: TACACTGCGGTGTGTGGTCATC, *PV* forward: CTGATGGCTGCTGGAGACAAAG, reverse: GAGATTGGGTGTTCAGGGCAGA, *SOX9* forward: AGGAAGCTCGCGGACCAGTAC, reverse: GGTGGTCCTTCTTGTGCTGCAC; *PRESTIN* forward: ATGGCTACCAGGTTGACGGCAA, reverse: CCTCCTGAACAAGGCTTCGAGA.

### Immunohistochemistry

Organoids were fixed with 4% paraformaldehyde. The fixed specimens were cryopreserved with a treatment of 30% sucrose and then embedded in tissue freezing medium OCT. Frozen tissue blocks were sectioned into 12 μm cryosections. For immunostaining, a 1% bovine serum albumin and 0.3% Triton X-100 solution was used for primary antibody incubation. FITC conjugated anti-rabbit IgG and CY3 conjugated anti-mouse IgG (Jackson ImmunoResearch) were used as secondary antibodies. A Hoechst stain was used to visualise cellular nuclei (Vector, VectaShield; http://vectorlabs.com/). Microscopy was performed on a Zeiss Axio Imager Z1 or Nikon Ti Eclipse confocal microscope.

The following antibodies were used: anti-SOX9 (rabbit, Millipore, 1:200, Polyclonal), anti-SOX2 (mouse, R&D Systems, 1:62.5, Clone 245610), anti-PAX2 (Rabbit, Thermo Fisher, 1:200, Polyclonal), anti-ECADHERIN (mouse, BD, 1:200, Clone 67A4), anti-ESPIN (rabbit, Novus Biologicals, 1:200, Polyclonal), anti-FACTIN (mouse, Abcam, 1:400, Clone 4E3.adl), anti-SYNAPTOPHYSIN (mouse, SIGMA, 1:200, Clone SVP-38), anti-ATOH1 (rabbit, Abcam, 1:200, Polyclonal), anti-GLUTAMINE SYNTHETASE (mouse, Millipore, 1:200, Clone GS-6), anti-TUBB3 (mouse, Covance, 1:500, Clone TUJ1), anti-MYO7A (rabbit, Abcam, 1:500, Polyclonal), anti-MYO7A (mouse, Santa Cruz, 1:100, Clone C-5), anti- NEUROFILAMENT (mouse, Invitrogen, 1:20, Clone RMdO-20), anti-CB2 (rabbit, Cayman Chemical, 1:50, Polyclonal), anti-OCM (rabbit, Novus Biologicals, 1:100, Polyclonal), anti-OTOP1 (rabbit, Novus Biologicals, 1:66.7, Polyclonal), anti-PRESTIN (rabbit, Thermo Fisher, 1:200, Polyclonal), anti-PARVALBUMIN (mouse, Thermo Fisher, 1:2000, Clone PARV-19). All antibodies were validated using embryonic tissues representative of the otic vesicle and foetal brain. Primary antibodies were omitted in control immunohistochemistry staining.

### FM1-43FX labelling

The presence of functional mechanosensitive channels was confirmed using a FM1-43FX dye uptake assay. Organoids were incubated in Advanced DMEM/F12 containing FM1-43FX (5 Μm final concentration; Invitrogen) for 1 min and then washed three times in Advanced DMEM/F12. Organoids were dissociated to single cells by 40 min incubation in Accutase then added Advanced DMEM/F12 to inactivate Accutase. The cells were then plated onto slides using the Cytospin method. For nuclear staining, Hoechst dye was used. The cells were imaged to confirm dye uptake and immediately fixed with 4% paraformaldehyde. For some experiments, cells were fixed and stained with otic lineage associated antibodies to confirm the identity of hair cells. Cells were examined using Nikon Ti Eclipse confocal microscope. Fields were chosen at random.

### Electrophysiological recordings

Organoids were transferred to the microscope chamber and immobilised using a nylon mesh fixed to a stainless steel ring. The chamber was continuously perfused with an extracellular solution containing (in mM): 135 NaCl, 5.8 KCl, 1.3 CaCl_2_, 0.9 MgCl_2_, 0.7 NaH_2_PO_4_, 5.6 D-glucose, 10 HEPES–NaOH, 2 Sodium pyruvate. MEM amino acids solution (from 50X concentrate), and MEM vitamins solution (from 100X concentrate) were added (Fisher Scientific). The pH was adjusted to 7.48 (osmolality ~308 mmol/kg). The organoids were observed using an upright microscope (Leica DM LFSA) with Nomarski differential interference (DIC) contrast optics, using a ×63 water-immersion objective. Whole-cell patch-clamp recordings were performed at room temperature using an Optopatch (Cairn Research) patch-clamp amplifier. Patch pipettes contained the following (in mM): 131 KCl, 3 MgCl_2_, 5 Na_2_ATP, 1 EGTA-KOH, 5 HEPES–KOH, 10 sodium phosphocreatine, pH 7.28 (osmolality ~295 mmol/kg). For recording sodium currents in isolation, patch pipettes contained (in mM): 137 CsCl, 2.5 MgCl_2_, 1 EGTA-CsOH, 2.5 Na_2_ATP, 10 sodium phosphocreatine, 5 HEPES-CsOH, pH 7.3 (osmolality ~292 mOsmol/kg). Patch pipettes were pulled to give a resistance in the range of 2–3 MΩ, and their tips were coated with surf wax (Mr Zogs SexWax) to minimise the fast capacitive transient across the wall of the patch pipette. Immediately before recording, the series resistance (*R*_s_) and the membrane capacitance (*C*_m_) of the cell were noted.

Data were acquired through a CED Power 1401 data acquisition interface using Signal software (Cambridge Electronic Design) and stored on a computer for off-line analysis using Origin (Origin Lab) data analysis software. For potassium current analysis, current amplitudes were measured from the steady-state current towards the end of the voltage step, before the tail currents emerged. For sodium current analysis, current amplitudes were measured at the peak of the sodium current.

## Electronic supplementary material

Figure S1

Figure S2

Figure S3

Figure S4

Figure S5

Figure S6

Figure S7

Supplementary figure legends
