# Supplementary figures and images for: Generating inner ear organoids containing putative cochlear hair cells from human pluripotent stem cells
Source: Cell Death Dis. 2018 Sep 11;9(9):922. doi: 10.1038/s41419-018-0967-1 (PMC6134051; doi:10.1038/s41419-018-0967-1)

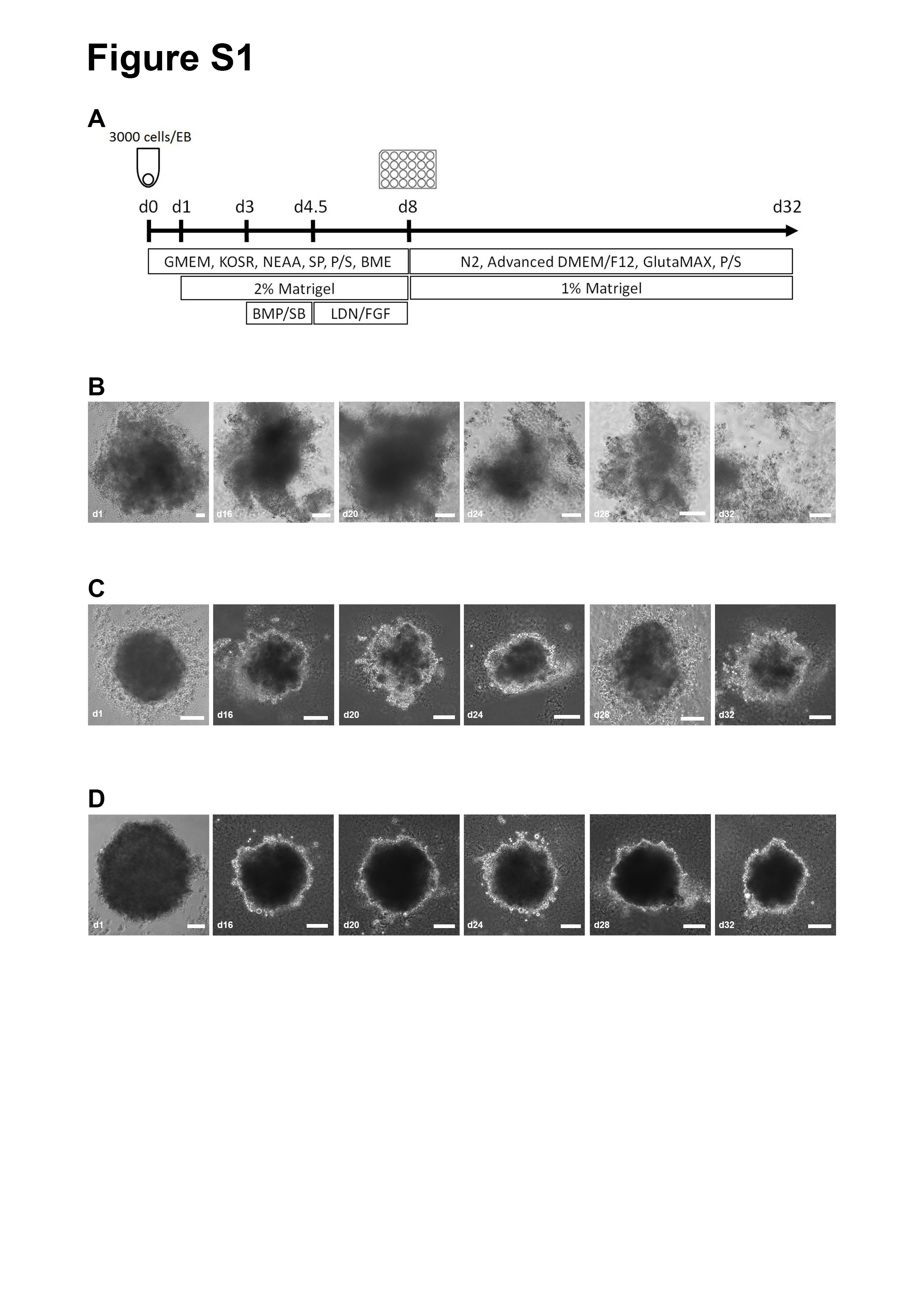

Supplement: Supplementary file 1 — Figure S1 [file 41419_2018_967_MOESM1_ESM.jpg]

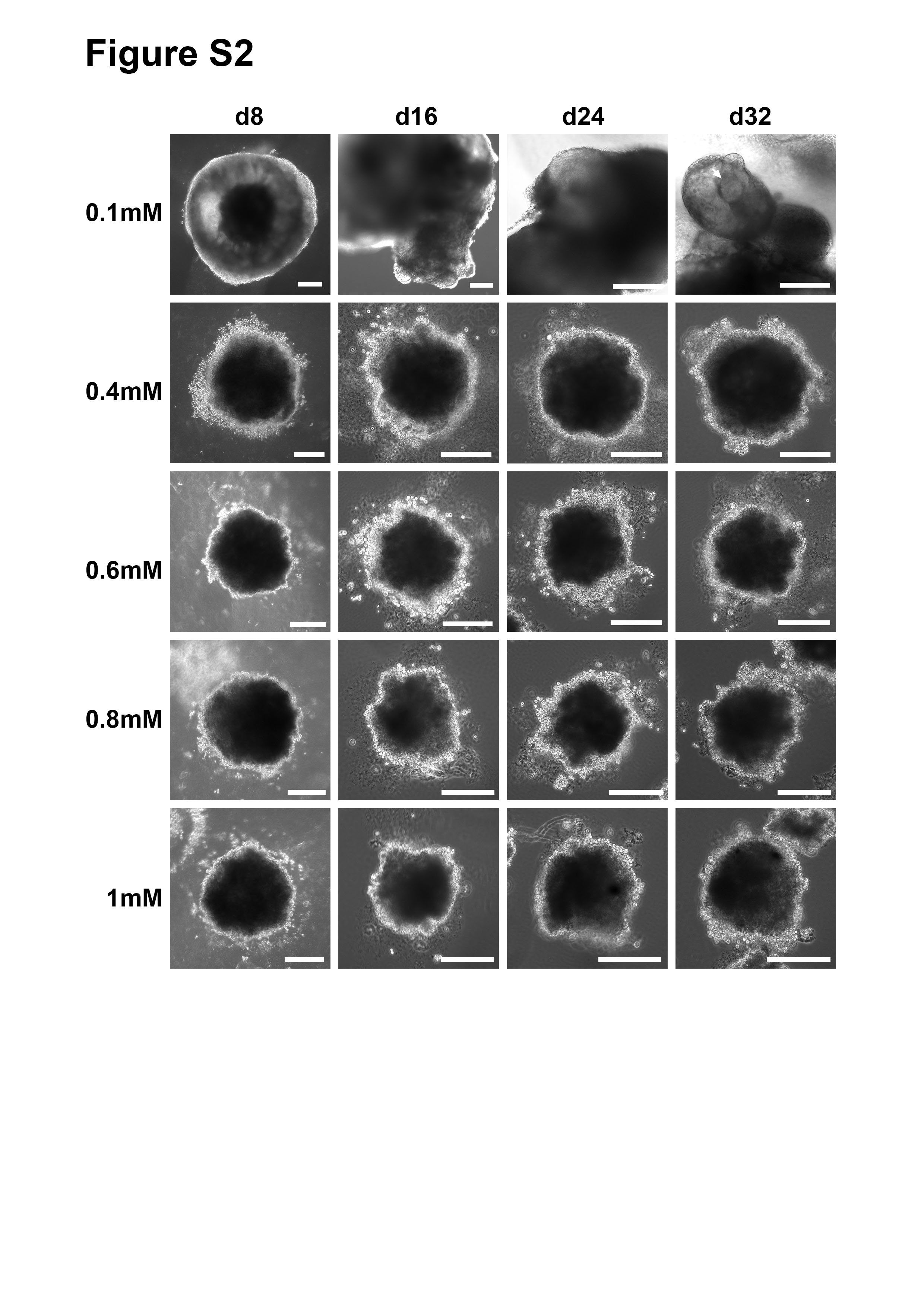

Supplement: Supplementary file 2 — Figure S2 [file 41419_2018_967_MOESM2_ESM.jpg]

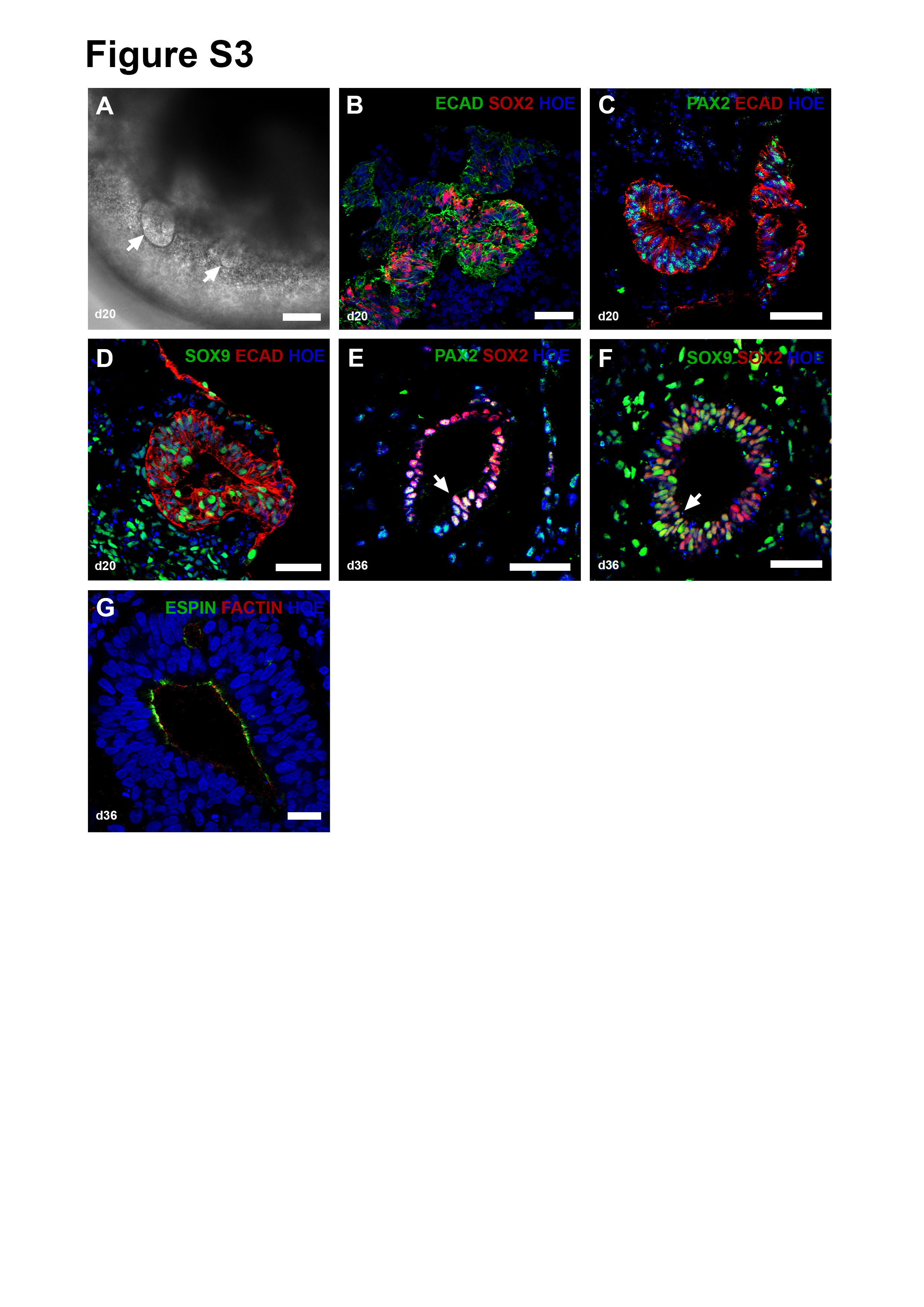

Supplement: Supplementary file 3 — Figure S3 [file 41419_2018_967_MOESM3_ESM.jpg]

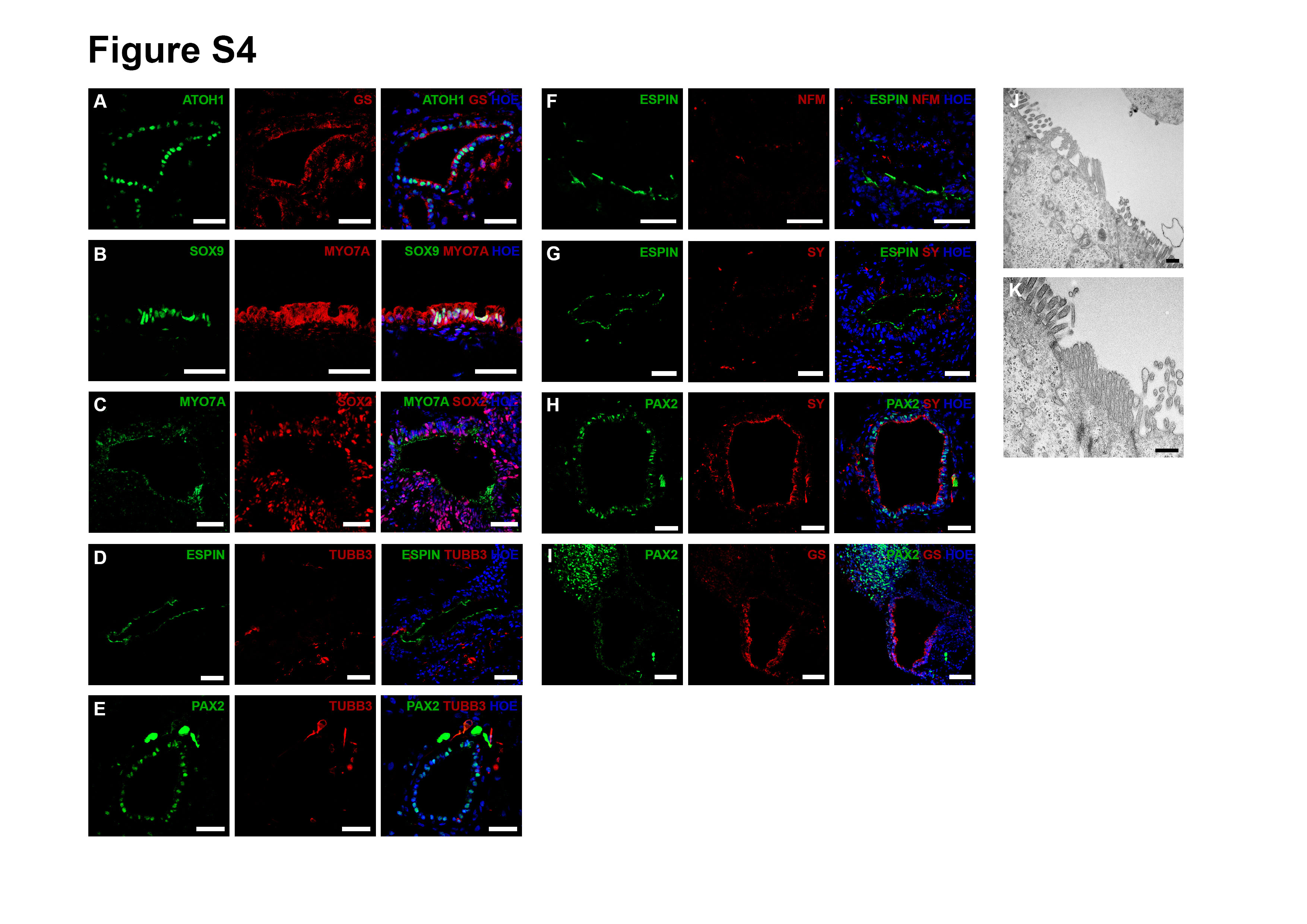

Supplement: Supplementary file 4 — Figure S4 [file 41419_2018_967_MOESM4_ESM.jpg]

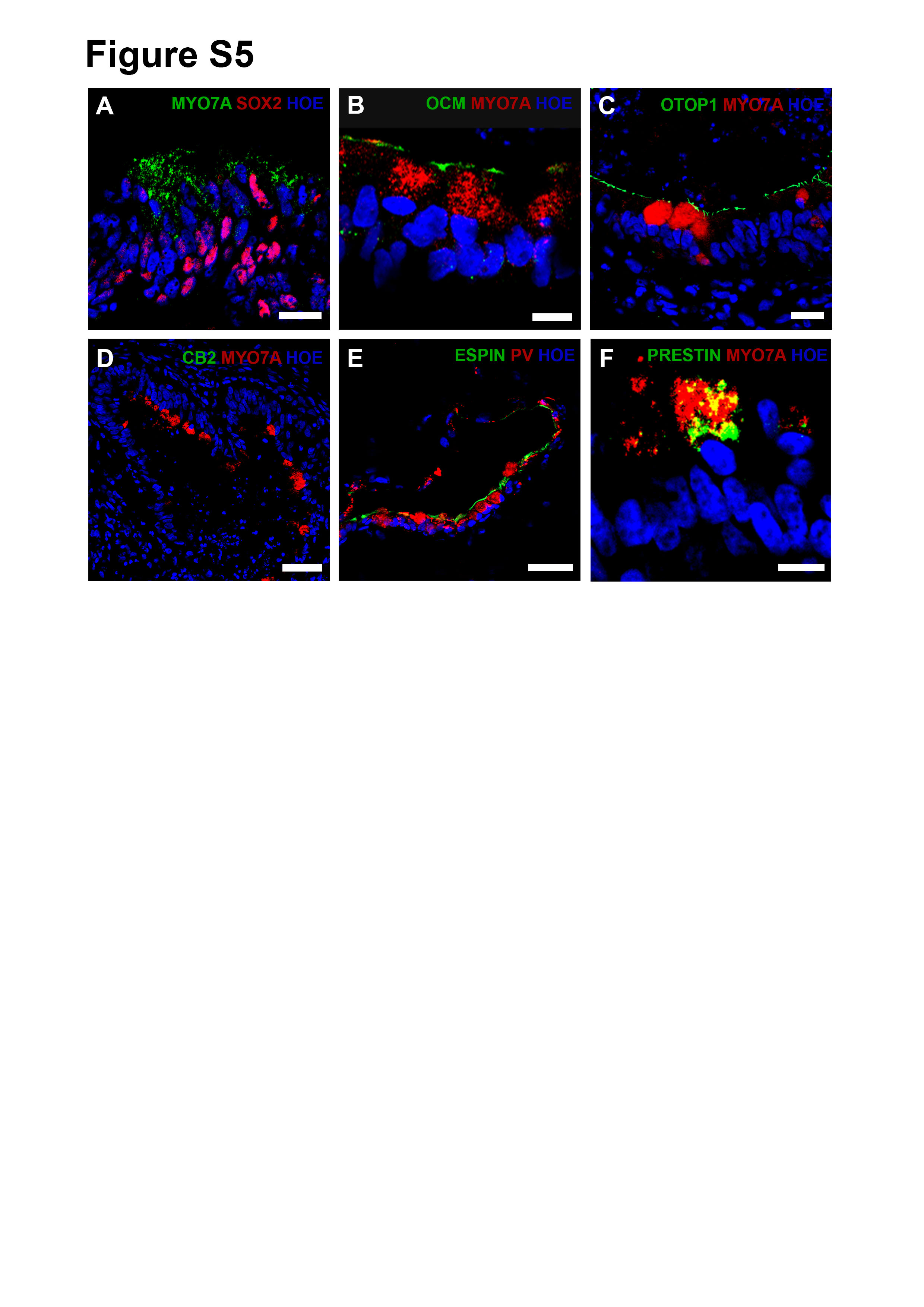

Supplement: Supplementary file 5 — Figure S5 [file 41419_2018_967_MOESM5_ESM.jpg]

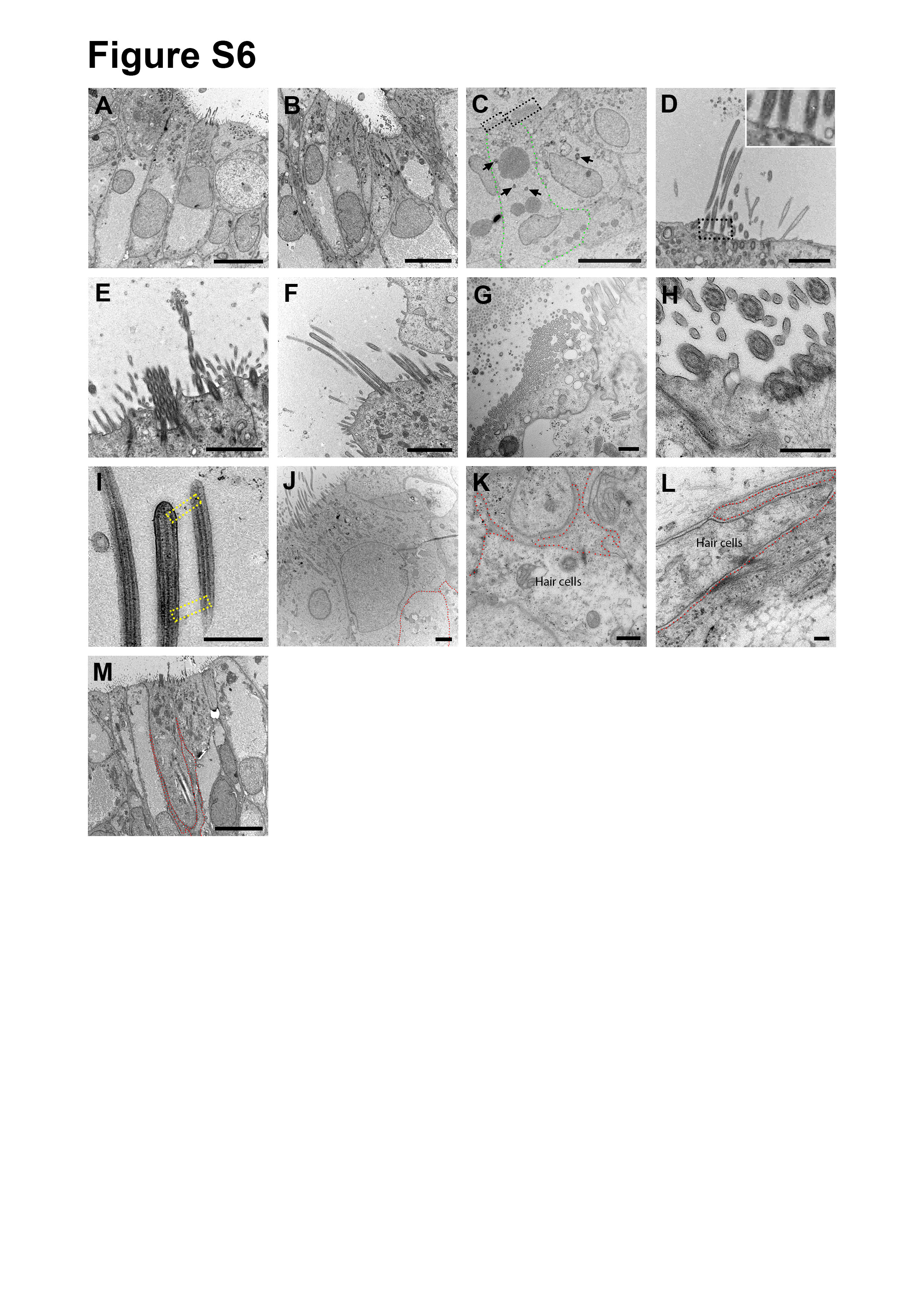

Supplement: Supplementary file 6 — Figure S6 [file 41419_2018_967_MOESM6_ESM.jpg]

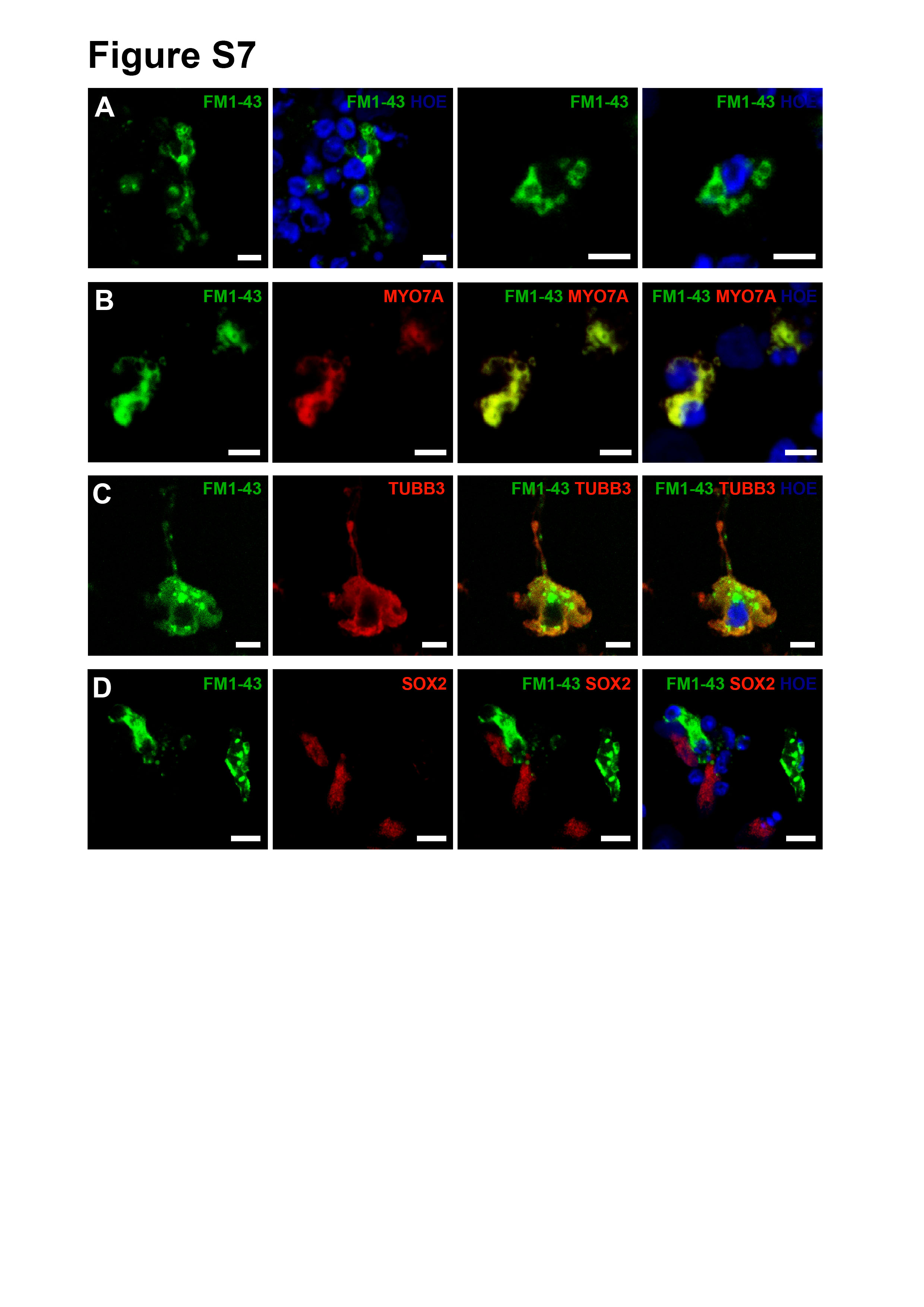

Supplement: Supplementary file 7 — Figure S7 [file 41419_2018_967_MOESM7_ESM.jpg]
