# Supplementary material for: Generating inner ear organoids containing putative cochlear hair cells from human pluripotent stem cells
Source: Cell Death Dis. 2018 Sep 11;9(9):922. doi: 10.1038/s41419-018-0967-1 (PMC6134051; doi:10.1038/s41419-018-0967-1)
Supplement: Supplementary file 8 — Supplementary figure legends [file 41419_2018_967_MOESM8_ESM.docx]

Supplementary information

**Figure S1:** **Application of Koehler’s protocol to hESC** (**A**) Schematic overview of the otic lineage guidance of mESC published by Koehler et al [^16^](#_ENREF_16). (**B**) Morphological changes during differentiation of hESC using the protocol published by Koehler et al [^16^](#_ENREF_16). (**C**) Morphological changes during differentiation of hESC using the culture conditions by changing the base media from GMEM to DMEM/F12. (**D**) Morphological changes during differentiation of hESC using 12000 cells per one organoid. Picture shown are representative of at least 3 independent experiments (n=3). Scale bars present 100μm (C, D), 50μm (B).

**Figure S2:** **Effect of 2-mercaptoethanol concentration on the formation of otic organoids**. Morphological changes during differentiation of hESC under the various concentrations of 2-mercaptoethanol showing otic vesicles (indicated by a white arrow) form only with a concentration of 2-mercaptoethanol of 0.1mM Scale bars present 200μm.

**Figure S3:** **Confirmation of differentiation towards otic cell types using an additional hiPSC line (SB-Ad3).** **(A)** By day 20, the organoids contain otic vesicles (white arrows) in the size range 100-200µm. (**B – D**) Immunohistological analysis of vesicular structures: (B) ECAD^+^ SOX2^+^, (C) PAX2^+^ECAD^+^, and (D) SOX9^+^ECAD^+^ in otic vesicles invaginating from the inner epithelium at day 20. **(E – G)** Immunohistological analysis of prosensory otic vesicles (day 36). The vesicles contain pro-sensory cells expressing PAX2/SOX2 (E), and SOX9/SOX2 (F) and possible pro-sensory cells start to express ESPIN and F-ACTIN (G). Data are representative of 8-16 organoids from at least 4 separate experiments (n=4). Scare bars represent 100μm (A), 50μm (B – G).

**Figure S4:** **Immunohistological and TEM analysis of hiPSC-derived otic vesicles at day 60.** ATOH1^+^ (**A**) and MYO7A^+^ (**B, C**) cells show possible hair cells and SOX9^+^ (**B**) or SOX2^+^ (**C**) are possible supporting cells. TUBB3^+^ putative neurons extend to possible sensory cells expressing ESPIN (**D**) and PAX2 (**E**). NFM^+^ bipolar neurons present near the sensory epithelium expressing ESPIN (**F**). SY^+^ synaptic neurons locate inside or near the sensory epithelium expressing ESPIN (**G**) and PAX2 (**H**). GS^+^ neurons innervate otic vesicles containing possible hair cells expressing ATOH1 (**A**) and PAX2 (**I**). Data are representative of 8-16 organoids from at least 4 separate experiments (n=4). **(J, K)** TEM of possible stereociliary bundles on day 60. Scale bars present 100μm (I), 50μm (A – H), 500nm (J, K).

**Figure S5: Immunohistological analysis to identify cell types derived from hiPSC at day 90.** MYO7A^+^ and SOX2^+^ show the cochlear hair cells and supporting cells, respectively (**A**). OCM^+^MYO7A^+^ cells may have an outer cochlear hair cell phenotype, while OCM^-^MYO7A^+^ cells are possibly inner cochlear hair cells or type II vestibular hair cells (**B**). OTOP1^+^ cells are possible vestibular supporting cells (**C**), but an expression of type II vestibular hair cell marker CB2 cannot be detected (**D**). PV^+^ cells are probably cochlear hair cells containing ESPIN^+^ stereocilia (**E**) and PRESTIN^+^MYO7A^+^ cells (**F**) may be outer cochlear hair cells. Data are representative of 16 organoids from at least 3 separate experiments (n=3). Scale bars present 50μm (D, E), 20μm (A, B, C), 10μm (F).

**Figure S6: Analysis of the morphologies of putative cochlear cell by TEM.** TEM of cylindrical shaped (**A, B**) and bulbous (**C**, green dots) hair cells containing possible presynaptic bodies (black arrows) and stereocilia (dotted square). Possible stereociliary bundles (**D, E, F**) and cross sections of the stereociliary bundles (**G**) and typical kinocilia (**H**). The yellow dotted square indicates putative tip links between stereocilia (**I**). Most of the possible hair cells make a connection with button like putative neurons (**J, K, L**, red dotted area) and sometimes with calyx nerve endings (**M,** red dotted area). Data are representative of 8 organoids from at least 4 independent experiments (n=4). Scale bars represent 10μm (A, B, C, M), 2μm (D, E, F, J), 500nm (G, H, I, K), 100nm (L).

**Figure S7: Functional properties of hiPSC-derived hair cells and neuronal cells using a FM1-43FX uptake assay at day 90.** Confocal images of dissociated otic organoids reveal FM1-43FX labelled cells (**A**). FM1-43FX labelled cells also express the hair cell marker MYO7A (**B**), neuronal marker TUBB3 (**C**), but not the supporting cell marker SOX2 (**D**). Data are representative of 8 dissociated organoids from at least 3 independent experiments (n=3). Scale bars present 10μm (D), 5μm (A, B, C).
